# Reliability of blood lactate as a measure of exercise intensity in different strains of mice during forced treadmill running

**DOI:** 10.1371/journal.pone.0215584

**Published:** 2019-05-03

**Authors:** Simon Lønbro, Jennifer M. Wiggins, Thomas Wittenborn, Pernille Byrialsen Elming, Lori Rice, Christine Pampo, Jennifer A. Lee, Dietmar W. Siemann, Michael R. Horsman

**Affiliations:** 1 Dept. of Experimental Clinical Oncology, Aarhus University Hospital, Aarhus, Denmark; 2 Dept. of Public Health, Section for Sports Science, Aarhus University, Aarhus, Denmark; 3 Dept. of Radiation Oncology, College of Medicine, University of Florida, Gainesville, FL, United States of America; Nottingham Trent University, UNITED KINGDOM

## Abstract

Exercise has long been known to be beneficial to human health. Studies aimed at understanding the effects of exercise specifically focus on predetermined exercise intensities defined by measuring the aerobic capacity of each individual. Many disease models involving animal training often establish aerobic capacity by using the maximal lactate steady state (MLSS), a widely used method in humans that has frequently been used in rodent studies. The MLSS is defined as the highest exercise intensity at which blood lactate concentration remains constant and is roughly equivalent to 70–80% of maximal aerobic capacity. Due to our up-coming experiments investigating the effect of different exercise intensities in specific strains of tumor-bearing mice, the aim of the present study was to determine the MLSS in athymic nude (NCr nu/nu and NMRI), CDF1, and C3H mice by treadmill running at increasing speeds. However, despite thorough exercise acclimation and the use of different exercise protocols and aversive stimuli, less than half of the experiments across strains pointed towards an established MLSS. Moreover, gently prodding the mice during low to moderate intensity running caused a 30–121% (p<0.05) increase in blood lactate concentration compared to running without stimulation, further questioning the use of lactate as a measure of exercise intensity. Overall, MLSS is difficult to determine and large variations of blood lactate levels were observed depending on the exercise protocol, mice handling strategy and strain. This should be considered when planning experiments in mice using forced exercise protocols.

## Introduction

The physiological benefits of exercise have been widely established for the prevention and management of numerous human diseases [1,2]. Exercise regimens elicit a dose-dependent response in terms of intensity, frequency, and duration, with variable exercise intensities inducing distinctive metabolic profiles [3,4]. Therefore, prescribed exercise interventions in the clinic need to be clearly and carefully defined in terms of routine, frequency, and intensity.

Maximal lactate steady state (MLSS) is one of the many established measurements for exercise intensity in humans [5,6]. The MLSS is defined as the maximal exercise intensity at which blood lactate concentration remains constant [7]. Beyond this exercise intensity, when anaerobic metabolism predominates, lactate production exceeds the rate of clearance and accumulates in the circulation. Thus, the MLSS has been has been used as a standard method for identifying the transition between aerobic and anaerobic metabolism [8].

Pre-clinical models of exercise physiology are often used to test the potential efficacy of exercise interventions in a variety of disease settings. There are various types of exercise regimens used in pre-clinical models, each with its advantages and disadvantages. In order to standardize exercise intensity and deliver a precise exercise dose, investigators use forced exercise models such as treadmill running.

Blood lactate concentration has frequently been measured in various rodent exercise studies with threshold values used as an indicator of exercise intensity [7,9–13]. However, mice show inter-strain variations in voluntary running distance and frequency [14,15] and maximal running speeds [16], which suggests that different strains have different characteristics concerning energy metabolism during exercise [17]. This highlights the need to establish the lactate threshold as an appropriate measure of exercise intensity and demonstrate that it can be used to design strain-specific exercise regimens [10,18]. A recent short review of the literature [13] identified 33 studies that had investigated the transition from aerobic to anaerobic metabolism during either incremental or continuous exercise by measuring different metabolites (such as creatine kinase or lactate), oxygen consumption or ATP production. Most of the studies investigated in this short review were conducted in rats and very few were performed in mice. Compared to the paucity of data measuring the anaerobic threshold in mice, a great number of studies focus on physical activity without measuring their exercise capacity and no regard for strain variation [16], making it difficult to compare data between research groups. By identifying an appropriate exercise intensity biomarker and establishing strain-specific characteristics, it would be possible to determine the effects of different exercise intensities in various models.

The purpose of this study was to determine if the lactate threshold, as defined by measuring the MLSS, was useful in establishing appropriate exercise regimens in mouse strains commonly used in pre-clinical oncology research. In view of our own tumor studies and the lack of published exercise data on these particular strains, we sought to determine the MLSS in athymic nude (NCr nu/nu and NMRI nu/nu), CDF1 and C3H mice. We examined blood lactate concentrations in mice before and after treadmill running using the classic multiple-day, constant-load MLSS protocol [19,20]. Given the paucity of studies clearly describing the methodology in establishing MLSS in these strains, we employed an explorative approach to establish a reliable exercise protocol that could allow us to identify MLSS across all strains. Ultimately, complete data from all the different experiments are provided in this manuscript.

## Methods

### Ethical approval

All animal studies were conducted according to the animal welfare policy of Aarhus University (http://dyrefaciliteter.au.dk), with approval of the Danish Animal Experiments Inspectorate as well as the Institutional Animal Care and Use Committee (IACUC) of the University of Florida (http://iacuc.ufl.edu).

### Animal models

The experiments were performed in female athymic NCr nu/nu (age 7–10 weeks; Taconic Biosciences, NY), female NMRI nu/nu mice (age 30 weeks; Janvier Labs, France), male C3H mice (age 16 weeks; Janvier Labs) and male and female CDF1 mice (age 16 weeks and 24 weeks respectively; Janvier Labs). All mice were maintained in a pathogen-free facility and provided with water and food ad libitum. The mice strains used in the experiments are frequently used in oncology research. Specifically, athymic nude mice and CDF1 females represent models for breast cancer research [21,22] and both CDF1 male and C3H male mice are used in pre-clinical colon cancer research [23]. Epidemiological data has shown that the risk of developing several cancers including both breast cancer and colorectal carcinoma risk can be reduced by physical activity [24].

### Blood sampling and lactate measurements

Blood lactate concentration was measured with the Lactate Plus handheld blood lactate meter, (Nova Biomedical, Waltham, MA, US) from mice at rest and immediately after exercise. As adapted from lactate measurement protocols [7,25] one drop of blood was collected via tail vein puncture onto a disposable strip for lactate analysis. Vein puncture was preferred due to its accessibility in mice. Additionally, a recent study in humans [26] suggests that arterial blood lactate and venous blood lactate can be used interchangeably as long as the lactate measurements are taken at least 10 minutes after the start of the exercise bout. Prior to measurements in the resting condition, all mice (except NCr athymic nude mice) were physically restrained for 30 minutes in plastic containers to ensure that all mice were as physically inactive as possible. Data from our lab showed that this 30-minute pre-conditioning lowered circulating lactate levels when compared to blood samples collected without pre-conditioning (data not shown). Initial experiments on NCr athymic nude mice were performed in a standardized, quiet and calm atmosphere. All animals were acclimated to restraint and blood sampling procedures. Starting three days before blood sampling, mice were restrained for 30 minutes daily followed by a tail pinch to mimic the sampling procedure. Immediately after the exercise bout, mice were restrained, and blood samples drawn within 2 minutes. Reproducibility of the lactate measurements was determined using test-retest measurements on 5–6 mice. The Pearson´s r between two succeeding lactate measurements at rest was 0.43, however this was significantly improved to 0.92 when the mice were acclimated daily for 3–4 days prior to the restraint and lactate measurement procedure (tail pinch). Similar correlations were found between two succeeding post-exercise measurements (r = 0.98). The correlation between two day-to-day (24 hours apart) resting measurements with preceding acclimation was 0.55.

### Statistical analysis

The sample sizes of all experiments were selected based on evidence from existing relevant literature in rodents investigating changes in lactate during incremental exercise protocols [7,13,16]. One way-ANOVA with repeated measures and post-hoc Student´s t-tests were used to investigate differences in lactate concentration at the various running speeds. Normal distribution was checked visually on all data using q-plots and histograms. All statistical analyses were performed in Stata (Stata 13, StataCorp, Texas, US). Data are presented as means (± 1 standard error of the mean) and outcomes of the statistical analyses are presented with p-values (alpha level of 0.05) and 95% Confidence Intervals.

### Exercise protocols and experiments

All exercise experiments were performed on an electrical treadmill designed for rodents (1055-SRM-E58 Exer-3/6, Columbus Instruments, Norfolk, UK and 1050-RM Exer-3/6 Columbus Instruments, Ohio, U.S.A.), with lane dividers ensuring mice ran individually with no interference. The treadmill speed was adjustable by 0.1 m/min. To reduce the amount of stress to the animals and ensure familiarization with the treadmill, all mice were subject to seven days of acclimatization. On day 1 animals were placed on a static treadmill. Subsequently, both speed (from 5 to 15 m/min) and duration (1 to 10 minutes) progressively increased over the following 6 days. The treadmill incline was adjustable by 5 degrees from 0–25 degrees. The inclines used in each experiment are reported below.

To establish lactate curves (blood lactate concentration as a function of running speed) and the MLSS we used the classic MLSS assessment protocol which measures the MLSS over the course of several days as described by Ferreira et al. [7], as opposed to the Palmer method [19,27,28].

Initially, 5 female athymic nude NCr mice were subjected to a 60-minute constant-velocity treadmill run at a 10-degree angle. All mice were acclimatized to the treadmill before the experiment. After acclimatization, all mice ran at increasing speeds with at least 24 hours between each run to ensure optimal recovery between runs. Ultimately, for each mouse, experimental days were typically done in 4–5 days which also reduced the chances of exercise induced physiological adaptations affecting lactate metabolism. At the beginning of each run the treadmill speed was slowly increased until the designated running speed was attained, and time was started. During each exercise bout in the initial experiments in the group of NCr nu/nu mice, puffs of compressed air were used to encourage mice to run. Mice that no longer responded to air puff stimulation were removed from the treadmill and blood lactate was immediately measured and recorded.

To address challenges with compliance (see results section) during the 60-minute protocol, subsequent experiments were reduced to 30 minutes using either 5 female NCr nu/nu mice or 5 female NMRI nu/nu mice. Additionally, NMRI mice were encouraged to run by gently prodding the hind legs and if this was ineffective, mice were gently placed on the platform, given a one-minute rest, and then placed back on the treadmill. The number of times mice were handled, also referred to as “paced”, during the exercise bout was recorded to assess the influence of handling on lactate values.

Despite reducing the duration of the exercise regimen, compliance was still a challenge and speculation that the mice were challenged in terms of motor skills due to the large running speed at 10-degree incline led to changes in the inclination of the treadmill to 25-degrees in subsequent experiments in 6 female nude NMRI mice, 6 male C3H mice as well as in both 5 female and 6 male CDF1 mice (comparing potential gender variations in blood lactate and MLSS establishment).

### Submaximal treadmill running with and without pacing

During all experiments mentioned above, a number of methodological challenges in maintaining compliance were encountered which required handling the mice, as well as, occasional gentle prodding. Repeatedly observed changes in blood lactate levels following handling and prodding both at rest and during low exercise intensities led to the hypothesis that handling the mice significantly affected lactate levels. To investigate this, we initiated parallel experiments with the purpose of investigating the effect of pacing on blood lactate levels during submaximal running across different strains of mice.

Over six days, with a single exercise bout performed per day, mice ran for 20 minutes at a fixed submaximal intensity on the same treadmill, with no incline. On Day 1 the speed was fixed at 12 m/min and all mice completed the entire exercise bout with no handling or pacing performed. Blood lactate was measured immediately post-exercise. On Day 2 the same group of mice were subjected to the same exercise bout but were constantly paced during the run. A standardized pacing regime was used in all experiments and consisted of prodding the hind legs and lifting the mice 18–22 times during the second half of the 20-minute exercise bout with an increasing frequency. Again, blood lactate was measured at the end of the exercise bout. Similar experiments (without and with pacing) were also performed at 15 and 20 m/min running speeds on 6 female and 6 male CDF1 mice as well as 5 female NMRI nu/nu.

## Results

### Experiments at 10-degree incline for 60 and 30 minutes

**Fig 1:**
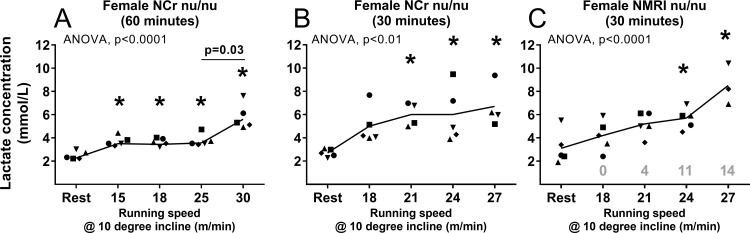
Blood lactate concentration (mmol/L) during rest and immediately post-exercise at increasing running speeds (m/min) in female nude NCr nu/nu mice (n = 5) at a 10-degree incline after 60 minutes of running **(1A)**; after 30 minutes of running in female NCr nu/nu mice **(1B)** and after 30 minutes of running in female nude NMRI mice **(1C)**. Points represent individual mice. 1A and 1B involved the use of air puffs to encourage mice to run, whereas 1C involved gentle prodding and physical handling. Statistical change (one-way ANOVA, p-value) in lactate concentration with increasing speed is displayed in each Fig. * indicates significant difference compared to the resting value (Student´s t-test, p<0.05). Specific p-value above line indicates significant difference compared to value at the previous running speed (Students t-test, p<0.05). If applicable, the mean number of times paced are reported above the x-axis.

In nude female NCr mice, the ANOVA analysis showed a change in lactate concentration with increasing running speed (p<0.0001) compared to resting state (Fig 1A). All post-exercise values were significantly higher than the resting value (Student´s t-test; P-values < 0.05). Blood lactate did not change between speeds of 15, 18 and 25 m/min. but increased significantly from 3.6 ± 0.2 mM at 25 m/min to 5.6 ± 0.5 mM at 30 m/min (p = 0.03; 95% CI: 0.4; 3.7). However, at the latter speed none of the mice completed the entire exercise bout. Air puffs were only used at 30 m/min where mice struggled and ultimately did not complete the run. These data suggest that the MLSS speed for this strain is around 25 m/min under this exercise regimen but without the completion of the exercise bout at 30m/min, the lactate threshold remains speculative.

In a subsequent experiment, to ensure that all mice were able to complete the prescribed exercise bout, the time was reduced to 30 min. To more closely identify the speed at which lactate increased beyond the MLSS, the nude female NCr mice ran at 18, 21, 24, and 27 m/min. According to the ANOVA, lactate values from running mice were higher than mice at rest (p<0.01; Fig 1B) and lactate concentration was significantly higher at all exercise intensities compared to rest (P-values<0.05). There was no difference in circulating lactate between mice running for 30 min at 18 to 24 m/min (p>0.05). The difference between lactate at 18 m/min versus 27 m/min was nearly significant (p = 0.06; 95% CI -0.1; 3.0). Thus, no clear MLSS was established during this experiment. It is important to note that at running speeds of both 24 and 27 m/min, two and three mice respectively, did not complete the entire exercise bout, since they refused to run and did not respond to continuous air puffs. Lactate was measured immediately after their removal from the treadmill.

The remaining mice completed the 30-minute bout of exercise. Non-compliant mice running at 24 m/min completed 20 minutes (designated as black dots and black squares) of exercise whereas the three non-compliant mice running at 27 m/min only completed 10 (designated as black dots), 14 (designated as black triangles), and 22 minutes (designated as black squares) each as shown in Fig 1B. All non-compliant mice displayed a slight increase in blood lactate, but no significant correlation was found. This observation shows that this group of mice had some difficulty running at speeds above 21 m/min despite no significant increase in blood lactate.

In the attempt to improve exercise compliance gentle prodding was employed instead of puffs of compressed air and pacing was used when prodding was ineffective. The experiment was repeated in five female NMRI nu/nu mice. Blood lactate concentration is illustrated in Fig 1C, showing a change in lactate levels with increasing speed (p<0.0001). Compared to the resting value, lactate levels trended higher at 18 and 21 m/min (p = 0.09; 95% CI: -0.2; 2.3 and p = 0.09; 95% CI:-0.5; 4.5, respectively) and significantly higher at 24 and 27 m/min (p = 0.01; 95% CI: 0.9;4.1 and p<0.0001; 95% CI: 4.7;5.1, respectively). Lactate concentration showed a slight rise from 5.7 ± 0.7 mM at 24 m/min to 8.5 ± 1.0 mM at 27 m/min (p = 0.09; 95% CI: -1.0;6.5), cautiously suggesting a MLSS at 24 m/min. All mice completed every exercise bout up to 24 m/min. However, the number of times mice were handled increased from 0 at rest and at 18 m/min to 4, 11 and 14 at 21, 24 and 27 m/min, respectively. Moreover, despite the adapted pacing strategy, none of the mice were able to run for more than a few minutes at 27 m/min. Many mice struggled above 18 m/min and attempts to improve compliance with rest periods and manual prodding were unsuccessful.

In summary, only one of three initial experiments provided data suggesting that, at or below 25 m/min, the MLSS is achieved. At 30m/min, though, the lactate increased, and mice were struggling to complete the exercise bout. However, in subsequent studies, mice struggled to complete 30 min exercise bouts below 25m/min without a rise in lactate, indicating that are other factors affect lactate levels.

Our experiments demonstrated that several mice were unable to run at the higher speeds for the entire exercise duration–some mice were unable to run for more than 3–10 minutes despite being able to run for 30 minutes at the previous speed with no change in blood lactate. However, as seen in Fig 1B, despite the increase in non-compliance and the constant use of air puffs from speeds 21 to 27 m/min, lactate values were unchanged. Previous unpublished data from our lab performed on CDF1 mice that showed no change in blood lactate after a 30-minute run at 27 m/min with no incline, led us to speculate that the mice were challenged in terms of their cadence and stride and not aerobically.

### Experiments at 25-degree incline for 30 minutes

As reported earlier [29], a lower cadence during running at a larger treadmill inclination may elicit a higher cardiorespiratory response and recruit a larger muscle mass that will eventually produce more lactate. To test whether running at a higher treadmill inclination would elicit a well-defined MLSS, we conducted a new set of experiments in six different female NMRI nu/nu mice, at a steeper incline of 25 degrees, which allowed for slower running speeds and potentially a lower cadence. Thus, similar experiments as described above at a 25-degree incline were performed at 12, 15, 17 and 20 m/min. Again, mice were encouraged to run by gently prodding their hind legs and placing the animal back on the treadmill belt after resting on the platform, as previously described.

**Fig 2:**
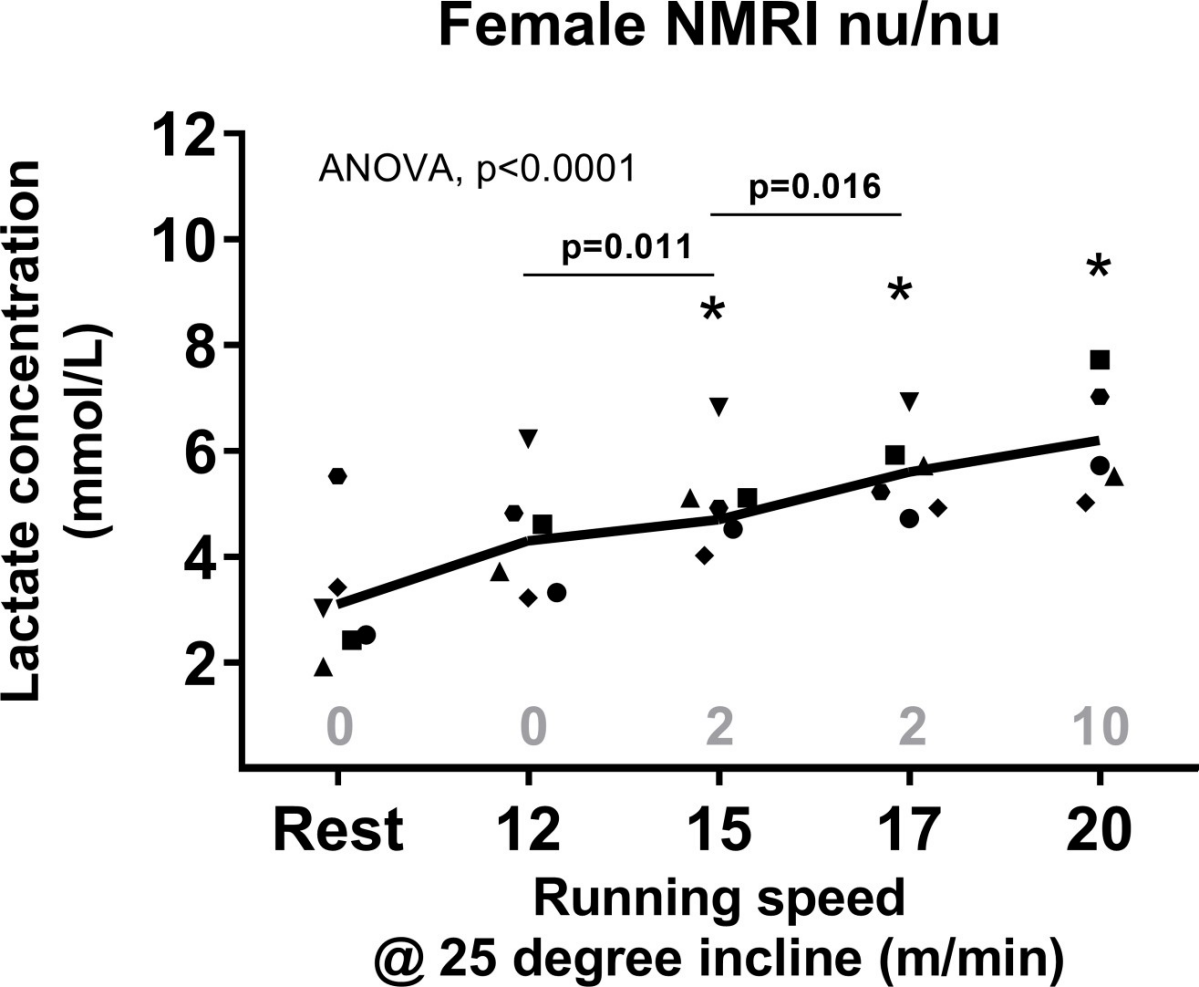
Blood lactate concentration (mmol/L) during rest and immediately post-exercise at increasing running speeds (m/min) in female nude NMRI mice after 30 minutes of running at 25-degree incline (n = 6). Points represent individual mice. Statistical change (one-way ANOVA p-value) in lactate concentration over time is displayed in each Fig. * indicates significant difference compared to the resting value (Students t-test, p<0.05). Specific p-value above line indicates significant difference compared to value at the previous running speed (Students t-test, p<0.05). The mean number of times paced are reported above the x-axis.

Fig 2 shows the changes in blood lactate concentration post-exercise at a 25-degree incline. Lactate concentration increased significantly with increasing running speed (p<0.0001) and all post-exercise values were significantly higher than the resting value (P-values<0.05) except when compared to the 12 m/min run (p = 0.11). Lactate concentration tended to increase from 5.3±0.2 at 17 m/min to 6.2±0.5 mM at 20 m/min (n = 5; p = 0.1; 95% CI: -0.3;2.1), not establishing a clear MLSS. The changes from 12 to 15 m/min, as well as from 15 to 17 m/min were significant, with an increase from 4.3±0.5 mM at 12 m/min to 5.1±0.4 mM at 15 m/min (p = 0.02; 95% CI: 0.3;1.3) and then to 5.6±0.3 mM at 17 m/min (n = 6; p = 0.02; 95% CI: 0.8;0.1). In agreement with our previous observations, mice were paced on average 10 times at 20 m/min compared to 15 and 17 m/min where mice were paced twice or compared to lower speeds where they were not paced at all. One mouse was accidentally injured during the exercise bout at 20 m/min and was excluded from the experiment and analysis.

**Fig 3:**
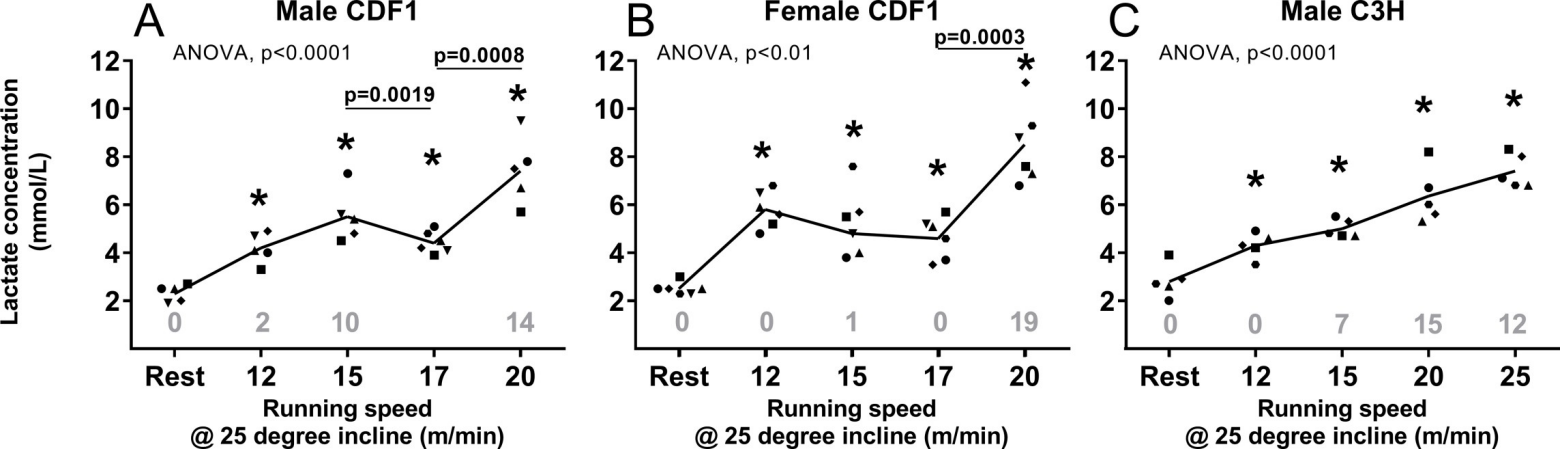
Blood lactate concentration (mmol/L) during rest and immediately post-exercise at increasing running speeds (m/min) in male (n = 5) **(3A)** and female CDF1 (n = 6) **(3B)** and male C3H (n = 5) **(3C)** mice after 30 minutes of running at 25-degree incline. Points represent individual mice. Statistical change (one-way ANOVA, p-value) in lactate concentration over time is displayed in each Fig. * indicates significant difference compared to the resting value (Students t-test, p<0.05). Specific p-value above line indicates significant difference compared to value at the previous running speed (Students t-test, p<0.05). The mean number of times mice were paced (lifted from the treadmill platform and placed back on the treadmill) are reported above the x-axis.

To determine MLSS in both male and female mice, a commonly used mouse strain in cancer research (CDF1) for both breast and colon cancer [23,30] was used (Fig 3A and 3B). Blood lactate concentration increased significantly with increasing running speeds for both male (Fig 3A) and female (Fig 3B) CDF1 mice (p<0.0001). All post-exercise values were significantly higher than resting blood lactate values. (P-values<0.01). In males, lactate levels increased significantly from 4.36±0.21 mM at 17 m/min to 7.44±0.63 at 20 m/min (p<0.01; 95% CI: 1.3; 4.8), suggesting an established MLSS speed around 17 m/min. Interestingly, lactate levels were significantly higher at 15 compared to 17 m/min (p = 0.02; 95% CI: 0.3; 2.0) and tended to be higher at 15 compared to 12 m/min (p = 0.08; 95% CI: -0.2;2.9). A similar difference between 17 and 20 m/min was observed for the females (p<0.01; 95% CI: 1.7; 6.0) with a significant increase from 4.6±0.4 mM to 8.5±0.7 mM, also suggesting an established MLSS speed at 17 m/min. There was no significant difference between the blood lactate concentration at 12 compared to 15 m/min or between 15 and 17 m/min (p>0.05). Of note, male mice were paced 2, 10 and 14 times on average at 12, 15 and 20 m/min respectively (pacing was not recorded at 17 m/min), whereas female mice were paced once at 15 m/min and 19 times on average at 20 m/min.

Subsequently, we tested another group of male mice in the C3H strain (used in prostate cancer research [31]) using the same exercise regimen. As shown in Fig 3C, the lactate concentration increased significantly with higher running speeds (p<0.0001) with all post-exercise values greater than resting blood lactate (P-values<0.05). However, despite a significant increase between 12 and 15 m/min (p = 0.03; 95% CI: -01; 1.3), lactate only tended to rise from 15 to 20 m/min (p = 0.07; 95% CI: -0.2; 2.9), as well as, 20 and 25 m/min (p = 0.07; 95% CI: -0.1; 2.2), making it difficult to establish a MLSS. The mice were paced 0, 7, 15 and 12 times at 12, 15, 20 and 25 m/min, respectively.

### Handling mice during exercise increased lactate levels

**Fig 4:**
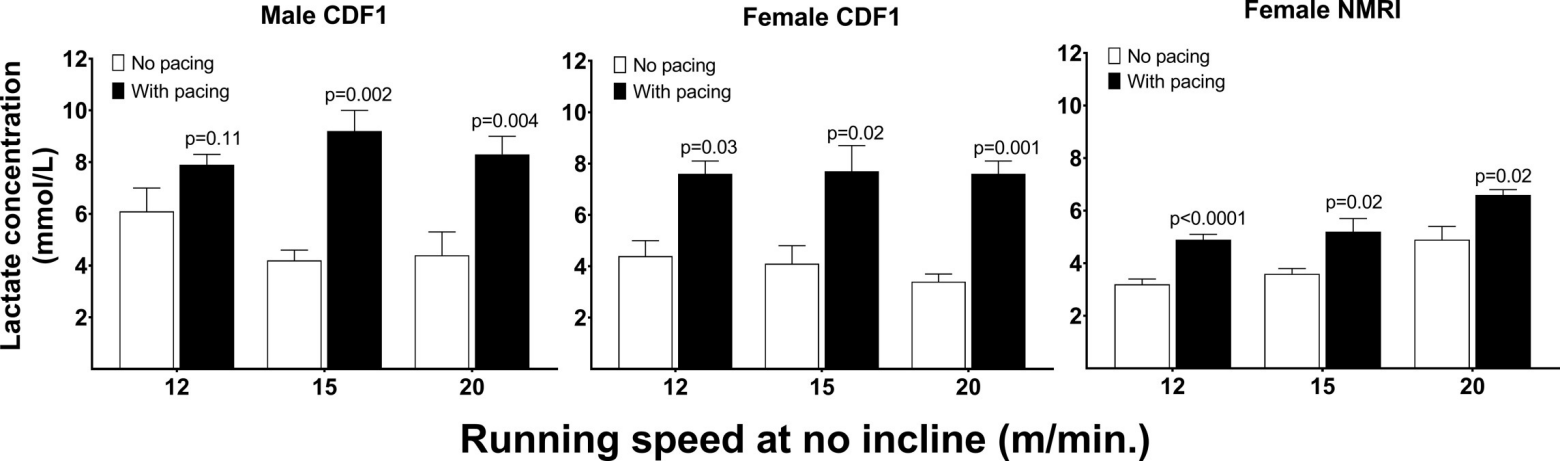
Mean (±SEM) blood lactate concentration (mmol/L) immediately post-exercise at different running speeds (m/min) in 6 male and 6 female CDF1 and 5 female nude NMRI mice for 20 minutes either without (designated with a white square) or with (designated with a black square) a standardized pacing regime. The specific p-values (Students paired t-test) report the statistical difference between blood lactate concentrations at the same running speed with and without pacing.

The experiments above led to the hypothesis that handling and pacing the mice during the exercise bout had a large influence on blood lactate concentration. To investigate this, we compared the influence of pacing on blood lactate, using CDF1 and NMRI nu/nu mice. As shown in Fig 4, the mice presented significant (Student’s paired t-test) increases in post-exercise lactate when paced at all submaximal running speeds when compared to exercise regimens at the same speed without pacing. Responses between gender and strain were comparable.

Male CDF1 mice showed post-exercise lactate levels of 6.1±0.9, 4.2±0.4 and 4.4±0.9 mM after running at 12, 15 and 20 m/minute, respectively, with no pacing. Pacing the same mice during the same exercise intensities increased the lactate values significantly by 30% (p = 0.1; 95% CI: 0.6; 4.2), 120% (p<0.01; 95% CI: 2.8; 7.2) and 90% (p<0.01; 95% CI: 2.0; 5.9) respectively, to 7.9±0.4, 9.2±0.8 and 8.3±0.7 mM. Similar findings were found in female CDF1 mice where pacing increased lactate levels by 71% (p<0.05; 95% CI: 0.5; 5.8), 90% (p<0.05; 95% CI: 0.8; 6.5) and 121% (p<0.01; 95% CI: 2.6; 5.7) from 4.4±0.6, 4.1±0.7 and 3.4±0.3 mM to to 7.6±0.4, 7.7±1.0 and 7.6±0.6 mM. In female NMRI mice lactate levels increased by 53% (p<0.001; 95% CI: 1.5; 1.8), 44% (p<0.05; 95% CI: 0.4; 2.7) and 35% (p<0.05; 95% CI: 0.4;3.1) from 3.2±0.2, 4.2±0.2 and 4.4±0.5 mM without pacing to 4.9±0.2, 5.2±0.4 and 6.6±0.2 mM with pacing.

## Discussion

The initial purpose of this study was to establish the MLSS in athymic nude (NCr and NMRI), CDF1 and C3H mice during constant velocity treadmill runs using the classical MLSS assessment protocol [19]. Data by Ferreira et al. show that minimal physical activity display a slight rise in blood lactate above baseline within the first 5 to 10 minutes of exercise and are kept constant throughout the exercise when conducted below MLSS intensity [9]. This increase from rest was observed in all our exercise experiments. At exercise intensities above the lactate threshold, blood lactate values post-exercise should rise above baseline and continue to rise with increased exercise load. However, despite thorough exercise acclimation protocols, the use of different exercise durations and different methods to encourage mice to run, less than half of our experiments across different strains suggested there was an established MLSS. Thus, we were unable to consistently establish MLSS curves across strains. We speculate that the mice may be either, near volitional exhaustion (as opposed to physical exhaustion) at the high speeds tested since the number of times they required prodding or pacing increased significantly, or the increased metabolic demands may be causing mice to struggle.

Our findings are in contrast to previous studies performed on C57BL/6J, FVBV/N and CD1 mice that have defined a clear MLSS. Indeed, the study by Ferreira in C57BL/6J reported a MLSS at a running speed of 15 m/min on a treadmill with no incline [7]. Billat et al. [16] established lactate threshold running speeds between 17.9–21.5 m/min in three different strains of mice (C57BL/6J, FVB/N and CD1) also using a forced treadmill running protocol. Neither of these studies included the strains of the present study commonly used for tumor growth, and we cannot rule out that the reason our results did not allow us to establish a clear MLSS could be due to differences in age, strain or the type of exercise and motivational strategy. The age of the mice in our experiments were chosen to mimic the age of the mice we normally use in studies on tumor-bearing mice, however mice availability in our lab caused practical challenges that increased variation between experiments, and this could affect the results. Given the age differences in our groups of mice (7 weeks up to 30 weeks of age), it would be expected to see a lower aerobic capacity in the animals aged 16 weeks or older, as observed by Ge and colleagues in C57BL/6 and CB6F1 mice [32]. We did not find a clearly defined MLSS in our older cohorts of mice, neither did we find a consistent result in our younger mice. Strain differences are evident in both treadmill and voluntary wheel exercises, where the same strain is unable to perform both tasks equally [16,18]. Hence, not only do mice have different aerobic capacities, but this is also dependent on the activity or exercise performed. In support of this significant inter-strain variability, our studies in various strains of mice suggest that the MLSS was around 17 m/min in male and female CDF1 mice (Fig 3A and 3B), whereas no significant rise in blood lactate was observed at higher intensities in NMRI nu/nu and C3H mice. However, it is important to note that the need for mechanical stimuli at higher speeds was increased among all mice, regardless of strain. Indeed, at the highest speed mice were mechanically stimulated 14 times on average, whereas at the previous speed they were encouraged only half as much (Figs 1, 2 and 3). This may indicate that despite the absence of an MLSS in both NMRI and C3H, all mice encountered a physical challenge at higher speeds. Based on our observations, we must consider the possibility that lactate above a certain value may be a hindrance to the ability of these mice to run. In our experiments, mice consistently struggled with the run and became non-compliant when blood lactate was above 5 mmol/L, irrespective of speed.

Moreover, the choice of motivational strategy may be an important factor that affects mouse metabolism and influences blood lactate concentration both at rest and during treadmill running. We hypothesize that activation of a stress response may be due to the animals loss of behavioral control, exhaustion and the use of stressful and novel motivational strategies [33]. A large number of forced exercise models in rodents are comprised of electrical shock grids at the rear of the treadmill that will discharge an electric pulse when the animal attempts to rest on the shock grid [16,18,29,34]. Using electrical shock as a motivational strategy during exercise not only produces a sudden emotional response, such as panic and anxiety [35] but it may also be harmful and cause injury to the animal if used at high voltage thus inducing metabolic changes not related to exercise [33]. Therefore, it is important to limit the use of shocks, by increasing familiarization to the environment and the treadmill. However, when in use, it is pertinent to thoroughly register the intensity of the stimulus (i.e., the voltage used), as well as, the number and frequency of shocks per exercise bout. The other two frequently used methods to encourage mice to run on a treadmill are compressed air puffs or mechanical prodding (with a stick or by manual stimulation) [33]. Initially, we used air puffs to encourage mice to run, however, despite the repeated use of air puffs mice no longer responded to this type of stimulation, hence we changed our method and applied mechanical prodding to encourage mice to continue running. Based on our data we are unable to confirm whether this approach, comprising gentle prodding or when necessary lifting the mice back on the treadmill when they rested on the platform, is less stressful than the use of electrical shocks.

To our knowledge, no previous studies have investigated the effect of different methods of motivational stimuli on blood lactate concentration after exercise and how these methods differ from each other in terms of affecting lactate concentration. Based on our experiments on NMRI and CDF1 mice (Fig 4), handling mice during the run has a significant effect on blood lactate. At low to moderate intensities, lactate concentration was 30–121% higher when the mice were manually stimulated compared to running without stimulation or handling. From our data, we are unable to elucidate the mechanisms behind this consistent increase in blood lactate, but we can assert that handling by gently prodding and handling the mice during the run had a substantial effect on blood lactate concentration. Previous data have shown that blood lactate may increase momentarily in response to stressful stimuli, such as an intraperitoneal injection in mice [36] or stressful psychosocial situations in humans [37]. In our experiments, we speculate that the increase in circulating lactate levels could be a result of elevated epinephrine cause by a stressful stimulus [38]. Hence, this strongly questions the use of blood lactate concentration as an appropriate indicator of exercise intensity during forced exercise protocols and alarmingly questions the validity of claiming an impervious association between exercise intensity and blood lactate concentration during forced exercise models in mice. In fact, in the study by Billat [16] up to 50 electrical stimulations were required to motivate mice to run to exhaustion, which, suggested by our data, may have influenced the lactate measurements reported. Unfortunately, detailed information was not provided on the number and frequency of the shocks used, so no comparison can be made between the animals that received electrical shocks and the ones that did not. Similarly, in the study by Ferreira and colleagues [7] the methods used to motivate mice to run were not disclosed. Directly comparing the blood lactate response between studies is largely problematic due to differences in motivational strategy (i.e. mechanical prodding vs. electrical stimulation) and also differences in strain, age and gender. This should be taken into consideration when interpreting results.

Indicators of stress during exercise have been reported previously by Contarteze et al. [39]. In that study, the authors reported a significant elevation in stress biomarkers (such as corticosterone and adrenocorticotrophic hormones) and increased blood lactate concentration in rats after exhaustive swimming and treadmill running compared to lower exercise intensities. In support, Moraska et al. found both positive and negative adaptations to forced treadmill exercise in rats, where the negative adaptations were related to increased stress levels (i.e., adrenal hypertrophy) [40]. Both of these studies applied electrical shock to motivate the animals to run and this may affect the physiological response to the exercise, however this remains speculative. Our observation that the reliability of the lactate measurements improved substantially when the mice were acclimated to the lactate measurement procedure also indicate that a stressful environment has a substantial impact on lactate concentrations, similar to findings by Thurston & Hauhart [36] who noted the increase after sham intraperitoneal injections.

To our knowledge, this is the first study providing evidence that questions the use of blood lactate concentration as a measure of exercise intensity and additionally presenting data on the effect of handling and pacing on blood lactate concentration during treadmill running in different strains of laboratory mice. This information is important since MLSS is often used in experiments involving a specific exercise intensity and thus, may affect the interpretation of lactate values in mice during forced exercise.

The classic multiple-day and constant-intensity testing protocol was chosen over a single-day incremental-intensity-testing protocol since the former has been proposed as the golden-standard MLSS protocol [20]. In addition, the data from the present study showing that handling the mice affects blood lactate concentration strongly questions the use of an incremental one-day protocol where continuous handling of the mice would be necessary and further stress to the animals would violate the reliability of the lactate measurements.

The test-retest reliability and the day-to-day variation of the lactate measurements of the initial experiments was less satisfactory (r = 0.43 and 0.55) and arguably a large measurement variation could hide the expected increase in lactate concentration and thus establishing the MLSS. However, following a thorough acclimation of all mice to the lactate measurement performed in the following experiments, the large test-retest variation was eliminated significantly improving the reliability of the measurements. Nonetheless, the substantial day-to-day variation and the disparity of results obtained after handling the mice strongly questions the use of MLSS as a valid measure of exercise intensity using the handheld blood lactate meter in mice.

Most experiments were performed between 08.00 AM-01.00 PM, for practical reasons and as circadian rhythm may affect metabolism and performance [41]. However, for reasons related to animal housing, the experiments performed on the nude NCr mice were done at the beginning of their active period in the evening. It cannot be ruled out that this may have affected their lactate levels, but the similar time of day of two different experiments and the diverging findings (Fig 1A and 1B) seem to reject this possibility.

## Conclusions and future perspectives

Despite several experiments in different strains of mice, we were unable to consistently establish MLSS curves in mice during treadmill running. These findings are in contrast to previously published studies in different strains of mice. Inter-strain variability may explain some of these discrepancies; however, this is only to some extent supported by our data. In addition, the experiments and diverging results led to the hypothesis that handling and pacing the mice during the exercise largely influences blood lactate concentration, which may in turn affect their compliance. This was confirmed in subsequent experiments showing increases in post-exercise lactate when paced at submaximal running speeds compared to exercise regimens at the same speed without pacing. The results presented here suggest that handling the mice during forced running induces an increase in blood lactate and interpreting the lactate increase exclusively as exercise-induced becomes highly problematic. In general, blood lactate level may be a useful measure to assess the metabolic profile at a specific exercise intensity and could roughly define the anaerobic contribution of the metabolism during exercise a given exercise bout. Despite the invasiveness, lactate measurements and analyses are simple, affordable and thus a highly available method. Nevertheless, as indicated by the present findings, establishing the MLSS is difficult and inconsistent in regards to different exercise -protocols, pacing strategies and strains. Thus, when planning intensity-regulated experiments in mice other means of grading exercise intensity should be considered. Direct measurements of oxygen consumption has frequently been used [42] and would provide a reliable measure of exercise intensity, however costs, personnel training and operation are more demanding. Furthermore, since gas analyses require a closed chamber surrounding the treadmill, the only plausible motivational strategy would comprise electrical stimulation that may cause stress to the animals and likely affect both metabolism and performance [43]. In conclusion, determining exercise intensity in forced exercise models in mice is difficult and further studies are needed to investigate the metabolic changes introduced by the methods per se and its consequences on performance.
